# Detection and characterization of *Candidatus* mycoplasma haemolamae haplotype in South American camelids farmed in Italy

**DOI:** 10.1007/s11259-025-11033-y

**Published:** 2026-01-20

**Authors:** Stefania Lauzi, Elisa Castaldo, Gabriele Ratti, Giulia Sala, Alessandra Cafiso, Alessia Facchin, Joel Filipe, Donatella Scavone, Cristina Crespi, Stefano Scarcelli, Laura Filippone Pavesi, Camilla Luzzago, Antonio Boccardo, Davide Pravettoni, Vincenzo Veneziano, Alessia Giordano

**Affiliations:** 1 Department of Veterinary Medicine and Animal Sciences, University of Milan, Via dell’Università 6, Lodi, 26900 Italy; 2 Department of Veterinary Medicine and Animal Production, University of Naples Federico II, Via Federico Delpino, 1, Naples, 80137 Italy; 3 Department of Veterinary Science, University of Pisa, via Livornese s.n.c, San Piero a Grado, 56122 Italy

**Keywords:** Alpaca, Llama, Anemia, Hemotropic *Mycoplasma* spp

## Abstract

*‘Candidatus* Mycoplasma haemolamae’ (CMhl) is a hemotropic bacterium infecting South American Camelids (SAC), whose epidemiology and clinical significance are still not fully elucidated. This study investigated CMhl by qPCR in blood samples of alpacas (*Vicugna pacos*) and llamas (*Lama glama*) in Italy in 2021–2024, characterizing CMhl haplotypes based on partial 16 S rRNA sequences and defining haematological findings in a subset of CMhl-infected animals. Statistical analysis was performed to detect potential risk factors associated with CMhl positivity. Out of 206 animals (200 alpacas and 6 llamas), CMhl was detected by qPCR in 88 (42.7%) animals. CMhl haplotype #1 and #2 were detected in 42.2% and 0.5% of the animals, respectively. Binary logistic regression analysis showed that higher CMhl positivity was significantly associated with larger herd size (*P* = 0.011), Northern Italy area of origin (*P* = 0.031) and warmer seasons (*P* = 0.003). The detection of two CMhl haplotypes confirms the high diffusion of CMhl in SAC farmed in Italy. Results confirm that CMhl usually causes subclinical infections, suggesting that antimicrobial treatment is likely not necessary based on qPCR positivity alone. Results focusing on the association of CMhl positivity with warm temperatures and large herd size suggest the need of further studies on the genetic features, epidemiology and immune response in CMhl-positive SAC from Italy, with emphasis on understanding potential vector transmission.

**Supplementary information:**

The online version contains supplementary material available at 10.1007/s11259-025-11033-y.

## Introduction

*‘Candidatus* Mycoplasma haemolamae’ (CMhl) is a hemotropic bacterium that infects several South American Camelids (SAC) including alpacas (*Vicugna pacos*) and llamas (*Lama glama*). To date the presence of CMhl has been reported in different countries and CMhl is considered an endemic pathogen of camelids. The first detections of CMhl in alpaca and llamas were from North America with reports from the US (McLaughlin et al. 1990; Almy et al. 2006; Lascola et al. 2009). Subsequently CMhl has been reported in different countries of South America, that represent the regions of origin of SAC, such as Peru, and Chile with infection also identified in vicunas (*Vicugna vicugna*) (Tornquist et al. 2010; Ramos et al. 2021; Gomez-Puerta et al. 2024). CMhl has also been identified in SAC from European countries, including the United Kingdom (Crosse et al. 2012), Switzerland (Kaufmann et al. 2011), Germany (Kaufmann et al. 2011; Wagener et al. 2024), Austria (Franz et al. 2016) and Finland (Wagener et al. 2024). CMhl has been reported also in SAC from New Zealand (van Andel et al. 2013; Dittmer et al. 2018). Besides alpaca and llamas, CMhl infection has been recently reported in dromedary camels (*Camelus dromedarius*) in Iran (Esmaeilnejad et al. 2019), Iraq (Matwari et al. 2022), Egypt (Eissa et al. 2024) and Somalia (Collere et al. 2025).

The transmission routes of CMhl remain unknown, however arthropods have been suggested as vectors for CMhl, as reported for other hemotropic mycoplasmas (Tornquist et al. 2010). In livestock, lice, flies, mosquitos, and ticks have been implicated in hemoplasma transmission (Arendt et al. 2024). In camels, molecular evidence has confirmed the potential role of ticks of the genus *Hyalomma* as vectors (Soliman et al. 2025). However, in many cases, evidence for vector-borne transmission for haemotropic mycoplasmas is circumstantial, as studies could not determine whether the detected DNA originated from the vector’s blood meal and thus belonged to the host, and lacks experimental confirmation (Millán et al. 2021). Other routes of transmission, such as vertical in utero transmission has been also suggested for CMhl (Pentecost et al. 2012).

Genetic characterization of CMhl has shown that the majority of CMhl sequences are closely related (Ramos et al. 2021). However, the presence of seven haplotypes based on 16 S rRNA sequences of CMhl has been reported in alpacas and llamas, and additional haplotypes have also recently been reported in vicunas from Peru (Ramos et al. 2021; Gomez-Puerta et al. 2024). CMhl haplotypes have shown different geographical distribution, and up to date only CMhl haplotype #1, has a worldwide distribution. CMhl haplotype #1 has likely emerged in South America and has subsequently been introduced to other countries, probably following the introduction of camelids from South America to these countries (Ramos et al. 2021). Once in the new geographic regions, haplotype #1 has likely evolved in the other haplotypes (Ramos et al. 2021). Although countries usually show a limited diversity of CMhl haplotypes, a high haplotype diversity has been observed in Germany, likely for the history of recurrent introduction in positive herds of new llamas and alpacas imported from other European or non-European countries (Neubert et al. 2021).

Various clinical manifestations ranging from subclinical infection to hemolytic anemia have been observed in camelids infected with CMhl. Clinical findings reported in association with CMhl infection seem to be non-specific. Among others, signs including lethargy, tachycardia, tachypnea, acidosis, depression, fever, azotemia, poor body condition due to chronic weight loss, pale mucous membranes and, infrequently, hypoglycemia have been reported (McLaughlin et al. 1990; Reagan et al. 1990; Almy et al. 2006; Crosse et al. 2012; Pentecost et al. 2012; Viesselmann et al. 2019). Despite infected animals are usually asymptomatic carriers that never develop clinical disease, deaths associated with high bacteremia may also occur (Tornquist et al. 2010). Severe and life-threatening cases of acute disease have mainly been reported in crias or pregnant female alpacas during gestation and parturition, or in conjunction with other concurrent diseases (McLaughlin et al. 1990; Messick 2004; Lascola et al. 2009; Meli et al. 2010). CMhl infection has been reported only occasionally or not at all in SACs that underwent necropsy, suggesting an unlikely major pathogenic role in these animals, even if pathological findings such as mycotic abomatitis, encephalomyelitis, fibrinous polysierositis, hemorrhagic enteritis, septicemia, polysierositis, interstitial pneumonia, splenic hyperplasia, or hydrotorax have been reported in CMhl-infected animals (McLaughlin et al. 1990; Clarke and Breuer 2022).

Studies on CMhl in Italy are limited and the genetic diversity of the bacteria has not been investigated so far. Thus, the aims of this study were *i*) to investigate the presence of CMhl and haplotypes in alpacas and llamas from different Italian regions, *ii*) to identify possible haematological alterations in CMhl-infected animals and *iii*) analyze the risk factors associated with CMhl infection.

## Materials and methods

### Animals and sampling

This study investigated CMhl presence by convenience sampling of 206 alpacas (200) and llamas (6) from Italy between November 2021 and June 2024.

Blood samples were collected from the jugular vein using vacuum tubes containing EDTA. Samples were submitted to the Veterinary Teaching Hospital (VTH) of Lodi for diagnostic purposes or as part of health checks. Upon arrival at the VTH, EDTA-blood samples were subjected to qPCR for the detection of CMhl DNA and haematological analysis was performed. According to the guidelines of the Institutional Animal Care and Use Committee of the University of Milan, formal approval was not required as blood samples were collected for diagnostic purposes or as part of health checks and with informed consent of the owners.

Data regarding sampling season period, herd of origin, signalment and, when available, clinical history were recorded for each animal. When available, data on the presence of ticks, at the time of sampling, were collected. Sampling season included spring (21 March – 20 June), summer (21 June – 20 September), autumn (21 September – 20 December), and winter (21 December – 20 March). Area of origin was categorized in North, Center and South Italy according to herd location. Herds of origin were categorized into small (≤ 20 animals), medium (> 20 and ≤ 50 animals) and large (> 50 animals). Animals aged < 6 months were classified as cria. Further age categories were weaners (≥ 6 months to < 1 year old), yearling (≥ 1 year to < 2 years old) and adult (≥ 2 years old) groups (Crosse et al. 2012; Castaldo et al. 2025). Animals were categorized according to clinical information, when available, in healthy and unhealthy. Animals were considered unhealthy in the presence of lethargy, stunted growth, cachexia or pale mucous membranes that may be caused by several diseases. Based on available haematological analyses results, animals were further categorized in the groups of animals showing haematological alterations and animals with parameters within normal range according to the reference values of the VTH. Specifically, animals were considered anemic when haematocrit and haemoglobin values were < 25% and < 11 g/dL, respectively; haemoconcentration was defined for haematocrit values > 38%; leukocytosis was defined for WBC count > 18.0 × 10^3^/µL; leukopenia was defined for WBC count < 5.0 × 10^3^/µL; thrombocytopenia was defined for platelet count < 100 PLT x10^3^/µL.

### Haematological analysis

Blood analysis was performed for samples submitted to the VTH lab within 24 h from collection (*n* = 59). Each blood sample was gently resuspended, and the complete blood cell count (CBC) was performed with the laser-based analyzer Sysmex XN-1000Vet (Sysmex Corporation, Kobe, Japan), using a specific setting created for alpacas.

On a subset (*n* = 34) of samples, a microscopic evaluation of a May-Grünwald Giemsa stained smear was performed to determine the presence of morphological peculiarities suggestive of the presence of CMhl; specifically, the observation at high power field magnification (1000X) of small coccoid basophilic structures, adherent to the erythrocyte membranes or freely present on the background were considered as consistent with the CMhl infection.

### DNA extraction

DNA was extracted from whole blood samples using a commercial DNA extraction kit (NucleoSpin Blood^®^ kit, Macherey-Nagle, Germany), following manufacturer’s instructions.

DNA pre-analytical quality control targeting vertebrate 12 S rRNA locus was performed on all samples (Kitano et al. 2007).

### Generation of positive control plasmids

Positive control plasmids were derived from a positive CMhl field sample previously obtained in our laboratory from an infected alpaca. A 600 bp of the 16S rRNA gene sequence of the bacterium was amplified by PCR (Meli et al. 2010) based on a 25-µL reaction containing 1x Phire Green Hot Start II PCR (ThermoFisher Scientific, Segrate –Milan, Italy), 1 µM of primer forward CMhlama.375f (5’-ACC ACG TGA ACG ATG AAG GTC-3’), 1 µM CMhlama.975r (5’-CGC AGT AGA TTA CAA GCC TTG GTA-3’) and 5µL of DNA. The amplicon was confirmed by Sanger sequencing (100% identity with CMhl, accession no. GU047355 and JF495171).

To obtain the positive plasmid control, a 72 bp of the16S rRNA gene of this CMhl-positive control sample was further amplified by PCR using 1 µM each of the forward primer Mhlama427f (5’-AAA AGC AGG ATA GGA AAT GAT TCT G-3’) and reverse primer Mhlama498r (5’-TGC TGG CAC ATA GTT AGC TGT CA-3’) (Meli et al. 2010), 1x Green GoTaq G2 Reaction Buffer (Promega, Milan, Italy), 0.2 mM dNTPs, 1.25U GoTaq G2 DNA Polymerase, and 5 µL DNA. The 72-bp amplified fragment of the positive control was run on agarose gel, excised, purified using a commercial kit (NucleoSpin Gel and PCR Clean‑up, Macherey-Nagel, Germany) following the manufacturer’s instruction and Sanger sequenced (Microsynth Seqlab, Germany) using the forward and reverse primers used for DNA amplification to verify the specificity of the reaction. The 72-bp amplified purified fragment was subsequently cloned into a pGEM-T Easy plasmid vector (Promega, WI, USA) using JM109 Competent Cells (Promega, WI, USA) following manufacturer’s instructions (Frackman and Kephart 1999) to produce a standard for the qPCR. Ten-fold dilutions of the plasmid between 10^9^ and 1 copies/µLwere subsequently prepared.

The amplification specifications (temperature and cycling conditions) of the PCR protocols are reported in Table S1.

### CMhl detection by real-time PCR

A real-time PCR (qPCR) targeting a portion of the 16S rRNA gene and amplifying a 72 bp fragment was performed according to Meli et al. (2010), using forward primer Mhlama427f (5’-AAA AGC AGG ATA GGA AAT GAT TCT G-3’) and reverse primer Mhlama498r (5’-TGC TGG CAC ATA GTT AGC TGT CA-3’) in a Sybr Green-based assay, to evaluate the presence of CMhl in the analyzed samples. The 20-µL qPCR reaction comprised 10µL of PowerUp Sybr Mastermix (ThermoFisher Scientific, Segrate –Milan, Italy), 0.5 µM of each primer and 5µL of DNA. The amplification specifications (temperature and cycling conditions) of the qPCR protocol are reported in Table S1. A blank control (DNAse-free water sample) was included in the qPCR reactions. Positive controls were represented by plasmids derived from a positive CMhl field sample previously obtained in our laboratory from an infected alpaca. The CMhl copy number in each sample was calculated based on the standard curve generated from 10-fold dilutions of the plasmid prepared between 10^9^ and 1 copies/µL. The amplification efficiency of the qPCR assay using the standard dilutions was 99.41% (slope of the curve − 3.32, R2 0.996, and Yintercept 41.9). The Tm range of the standard dilutions was 74.9–75.5 °C. The detection limit of the qPCR assay was 5 × 10^0^ copies.

### CMhl haplotype and phylogeny

Samples with qPCR-positive results were amplified by a nested PCR approach using a combination of two different PCR protocols targeting the 16S rRNA gene previously published (Meli et al. 2010). More precisely, 5 µL of DNA were used as template for the outer PCR reaction targeting the nearly complete (1420 bp) 16S rRNA gene based on a 25-µL reaction containing 1x Phire Green Hot Start II PCR (ThermoFisher Scientific, Segrate –Milan, Italy), 500 nM of primer forward CMhlama.5f (5’-GGA TTA ATG CTG GTG GTA TGC-3’) and 500 nM of primer reverse CMhlama.1424r (5’-CCA ATC AAA ATT ACC AAT CTA GACG-3’).

One µL of the PCR product was amplified in the inner reaction targeting a 600 bp fragment of the 16S rRNA gene based on a 25-µL reaction containing 1x Phire Green Hot Start II PCR (ThermoFisher Scientific, Segrate –Milan, Italy), 1 µM of primer internal forward CMhlama.375f (5’-ACC ACG TGA ACG ATG AAG GTC-3’) and 1 µM CMhlama.975r (5’-CGC AGT AGA TTA CAA GCC TTG GTA-3’). The amplification specifications (temperature and cycling conditions) of the PCR protocols are reported in Table S1.

The 600 bp amplicons of the 16 S rRNA were run on agarose gel, excised, purified using a commercial kit (NucleoSpin Gel and PCR Clean‑up, Macherey-Nagel, Germany) following the manufacturer’s instruction, and Sanger sequenced by a commercial sequencing facility (Microsynth Seqlab, Germany) using the same internal forward and reverse primers used in the inner round of DNA amplification. The sequence data were assembled using BioEdit software version 7.0 (freely available at http://www.mbio.ncsu.edu/BioEdit/bioedit.html). The sequences were then compared with those available in GenBank using BLAST (http://blast.ncbi.nlm.nih.gov/Blast.cgi). All the consensus sequences were aligned against the CMhl haplotypes retrieved from GenBank using Clustal X in BioEdit software v.7.0 to identify the haplotype of the strain.

For phylogenetic analysis, the sequences were aligned with CMhl reference sequences and other sequences of other *Mycoplasma* spp. retrieved from GenBank using Clustal X in BioEdit software.

v.7.0. Phylogeny was estimated by maximum likelihood (ML) method with 1000 bootstrap replicates using MEGA v.12 (Kimura 1980; Felsenstein 1981; Kumar et al. 2024). The model used for phylogeny based on ML method was chosen according to the lowest AIC value. The percentage of nucleotide similarity of pairwise evolutionary distances was calculated using MEGA v.12 for sequences comparison.

The two representative sequences from our work, namely CMhl alpaca 1 2021 Italy (haplotype 1) and CMhl alpaca 2 2023 Italy (haplotype 2), were submitted to GenBank with accession numbers PX520833 and PX520834, respectively.

### Statistical analyses

Pearson chi-square test or Fisher’s exact test were used to evaluate the differences between proportions of CMhl-positive animals and animal species, sex, age categories, area of origin, herd size, sampling season, health status and, when available, presence of haematological alterations.

The binary logistic regression was based on preliminary data of univariable analysis, testing associations between CMhl positivity and host- or management-related factors (e.g., sex, age, herd size, geographical area, season, health status, and hematological parameters when available) by Pearson’s chi-square or Fisher’s exact test, as appropriate. Variables with *p* < 0.3 in the univariable analysis and with variance inflation factors (VIF) < 3 were included in a binary logistic regression model to estimate odds ratios (OR) and 95% confidence intervals (CI). Multicollinearity among predictors was checked and model fit was assessed by the Hosmer–Lemeshow test.

Mean CMhl loads were calculated for CMhl-positive samples only, and were compared across categories of sex, age, and clinical status using Student’s t-test or one-way ANOVA, according to data distribution.

All statistical analyses were performed using SPSS version 28.0 (IBM Corp., Armonk, NY, USA) or Graph Pad Prism 9 software (GraphPad Software, La Jolla, CA, United States), and *p* < 0.05 was considered statistically significant, whereas tendency was considered in the presence of p-values > 0.05 but < 0.1.

Agreement between qPCR and blood smear was evaluated by Cohen’s Kappa statistic. Kappa value < 0.00 indicates a poor concordance, 0.00 to 0.20 a slight concordance, 0.21 to 0.40 a fair concordance, 0.41 to 0.60 a moderate concordance, 0.61 to 0.80 a substantial concordance, and ≥ 0.81 represents almost perfect concordance (Landis and Koch 1977). Sensitivity and specificity of blood smear were also calculated using qPCR as the reference standard (Trevethan 2017).

## Results

### Study animals and haematology

**Fig. 1:**
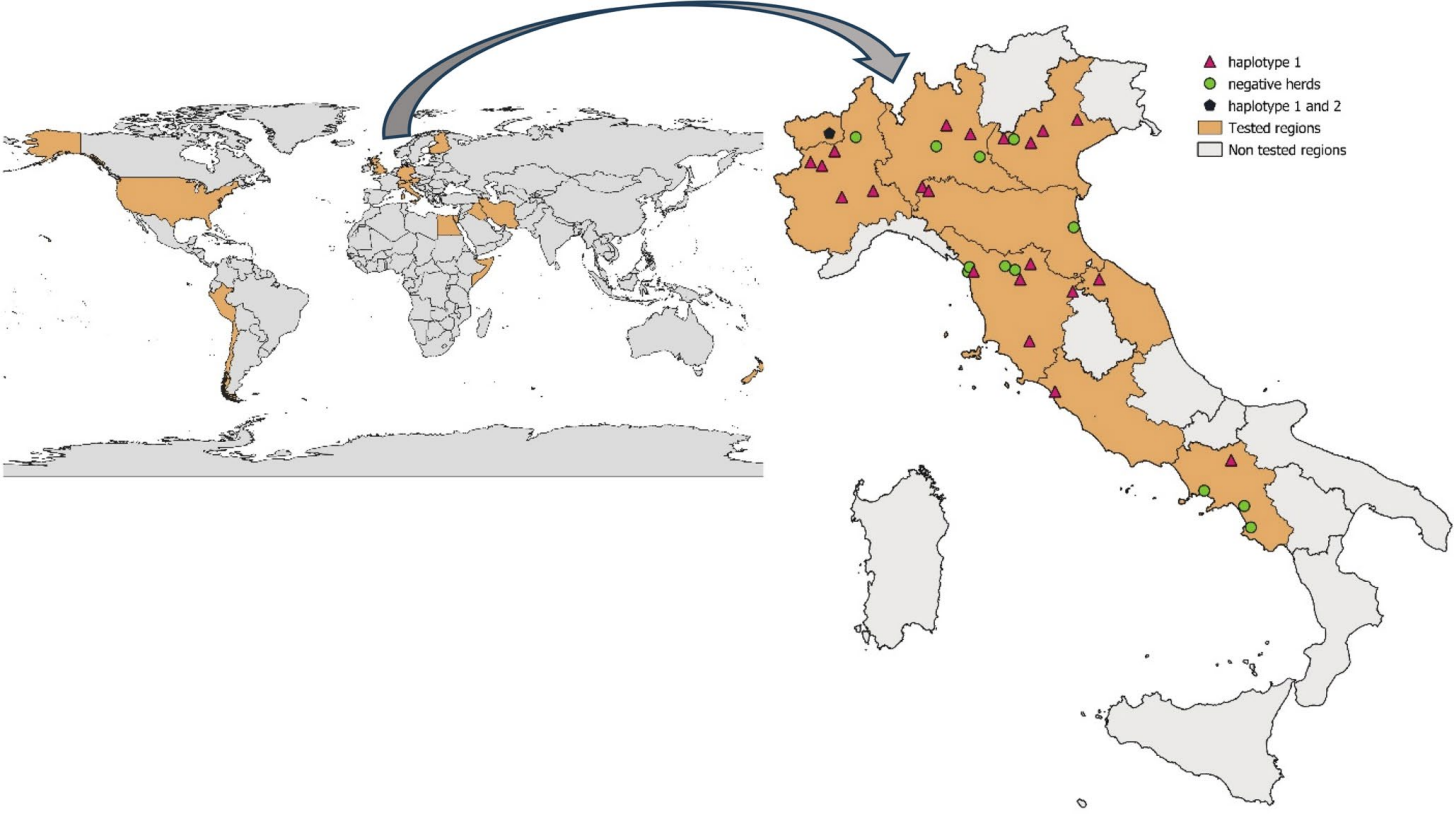
Geographical distribution of CMhl positivity reported in camelids worldwide (countries in orange) and in herds from Italy based on the results of this study

A total of 206 animals were tested for CMhl, including 200 alpaca and 6 llamas. The median age of animals was four years and three months, ranging from 25 days to 23 years of age. Animals were from 9 different Italian regions, covering North, Center and South Italy, and 35 different herds (Fig. 1). One herd only harbored both alpacas and llamas.

Data of the animals’ characteristics are reported in Table 1. Tick inspection was performed on only 26 alpacas. In these animals, no ticks were observed at the time of sampling, which was carried out during the spring or autumn seasons.

**Table 1:** Number and percentage of CMhl-positive alpaca and Llama according to characteristics of animals

Variable	Category	No. tested	No. CMhl-positive (%)	*P *value*
Species	alpaca	200	83 (41.5)	0.0858
	llama	6	5 (83.3)	
Sex	male	103	49 (47.6)	0.159
	female	103	39 (37.9)	
Age	cria (< 6 months)	13	1 (7.7)	**0.0043**
	weaner (≥ 6 months to <1 year)	15	11 (73.3)	
	yearling (≥1 year to <2 years)	34	17 (50)	
	adult (≥ 2 years)	144	59 (41)	
Herd size	small (≤20)	137	49 (35.8)	**0.003**
	medium (> 20 and ≤ 50)	61	32 (52.5)	
	large (>50)	8	7 (87.5)	
Area	North	75	35 (46.7)	**0.0374**
	Center	115	51 (44.3)	
	South	16	2 (12.5)	
Season	spring	70	19 (27.1)	**0.0039**
	summer	4	3 (75)	
	autumn	37	22 (59.5)	
	winter	95	44 (46.3)	
Health status**	clinically healthy	111	40 (36.0)	**0.0109**
	clinically unhealthy	14	10 (71.4)	
Haematology***	alteration	17	9 (52.9)	0.154
	normal	42	13 (31)	
Anemia***	yes	6	4 (66.7)	0.128
	no	53	18 (34)	
Leukocytosis***	yes	5	3 (60)	0.438
	no	54	19 (35.2)	
Haemoconcentration***	yes	3	1 (33.3)	1
	no	56	21 (37.5)	
Thrombocytopenia***	yes	4	3 (75)	0.4384
	no	55	19 (34.5)	

*P values based on Pearson chi-square test or Fisher’s exact test results to evaluate the differences between proportions of CMhl-positive animals and variables; ** Health status was available for 125 animals; ***Haematological parameters were available for 59 alpacas

Haematological data of these animals were available in 59 alpacas and are reported in Table 2.

**Table 2:** Results of haematological analysis in 59 alpacas

No. alpacas (%)	slight anemia	haemoconcentration	leukocytosis	leukopenia	thrombocytopenia
4 (6.8)	*+*	*-*	*-*	*-*	*-*
2 (3.4)	*+*	*-*	*+*	*-*	*-*
3 (5.1)	*-*	*-*	*+*	*-*	*-*
3 (5.1)	*-*	*+*	*-*	*-*	*-*
1 (1.7)	*-*	*-*	*-*	*+*	*-*
4 (6.8)	*-*	*-*	*-*	*-*	*+*
42 (71.2)	*-*	*-*	*-*	*-*	*-*

In 17 out of these 59 cases, slight haematological changes were detected. Specifically, six animals showed lower haematocrit, haemoglobin and RBC count compared to the reference interval consistent with a slight anemia; three showed increased erythroid parameters, consistent with haemoconcentration probably due to a slight dehydration; five animals had slightly increased leukocytes, consistent with stress or inflammation (and among these, two were also anemic); one animal was moderately leukocopenic and four had lower platelet counts consistent with thrombocytopenia. Haematological analyses were available for two out of the four unhealthy animals.

### CMhl occurrence and haplotype

**Fig. 2:**
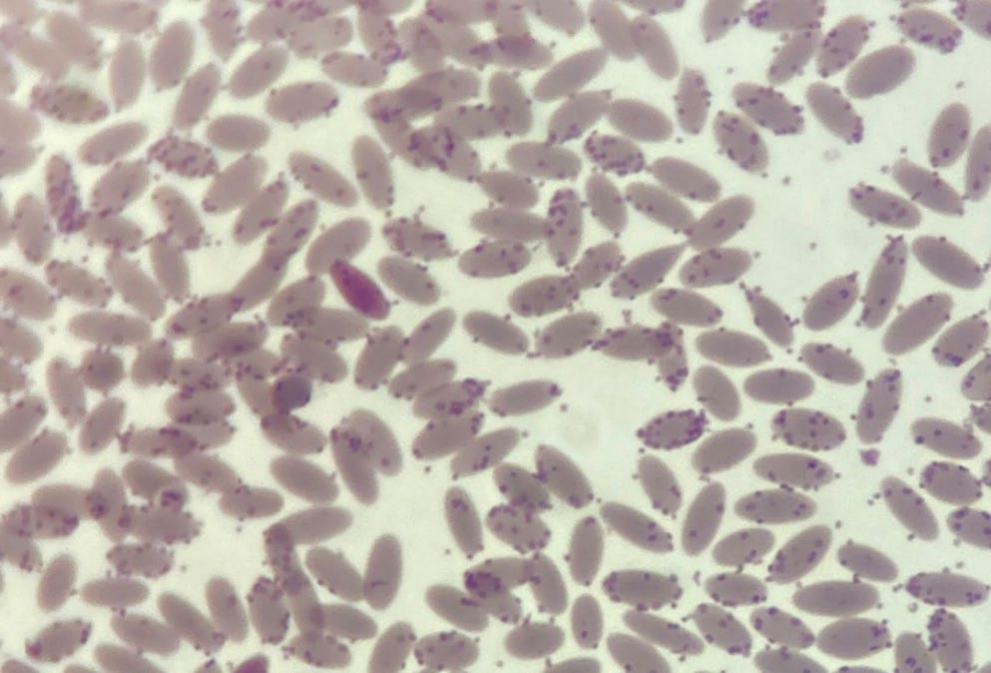
Peripheral blood smear stained with May-Grünwald Giemsa, 1000X (oil) magnification of a cria with a massive CMhl infection. The hemotropic bacteria appear as basophilic coccoid structures adherent to the erythrocyte membranes or as free on the background

Blood smears were performed in a subset of 34 samples and results suggestive of CMhl infection were detected in six (17.6%) animals, corresponding to the alpaca cria with the highest bacterial load of 1.9 × 10^9^ DNA copies/µL blood (Fig. 2) and animals with bacterial loads ranging from 2.9 × 10^6^ to 5.7 × 10^3^ DNA copies/µL blood.

The fragment of the vertebrate 12 S rRNA locus was successfully amplified in all alpacas and llamas blood samples (data not shown). CMhl was detected by qPCR in 88/206 (42.7%; 95% C.I: 36%−49.53%) animals, occurring in both alpacas (83/200; 41.5%; 95% CI: 34.7–48.3%) and llamas (5/6; 83.3%; 95% CI: 53.5–100%). Samples with qPCR-positive results showed Tm ranging from 74.9 °C to 75.5 °C. Results of CMhl-positive animals according to analyzed variables are reported in Table 1.

Results of qPCR showing CMhl positivity according to herd provenience are reported in Fig. 1 whereas results according to the herd size and area of provenience are reported in Table S2.

Among animals farmed in the 35 herds, the number of animals tested per herd ranged from one to 27 animals and the percentage of animals tested per herd ranged from 2.5% to 100%. CMhl was detected in at least one animal in 23/35 (65.7%) of the tested herds. In positive herds, CMhl positivity calculated on the total number of animals tested per herd ranged from 8.3% to 100%.

CMhl load in qPCR-positive animals ranged between 8 and 1.9 × 10^9^ DNA copies/µL blood and showed a mean value of 2.2 × 10^7^ and median value of 2.5 × 10^4^ DNA copies/µL blood. CMhl loads ≥ 1 × 10^6^ DNA copies/µL blood were reported in five out of the 88 (5.7%) CMhl-positive animals. The mean, median, minimum and maximum CMhl loads in CMhl-positive animals according to sex, age and health status are reported in Table S3. The animal with the highest CMhl load was the only CMhl-positive cria from this study. The mother of this cria was CMhl-positive but the cria had been rejected by its dam at birth and was subsequently fed with artificial bovine colostrum and pasteurized whole cow’s milk. The alpaca cria was admitted at the VTH for weakness and lethargy. Upon admission, the cria showed pale mucous membranes, tachycardia, and moderate dehydration. Blood tests revealed severe hypoglycemia (1.1 mmol/L) and mild regenerative anemia, with a hematocrit of 17% and hemoglobin level of 5.6 g/dL.

Results of the nested PCR used for sequencing showed positive results in 85/88 CMhl-qPCR positive samples. The three negative samples in the nested PCR showed low CMhl loads in the qPCR assay and could not be sequenced. Sequencing aimed at CMhl haplotype identification was successfully obtained from the 85 samples that were positive in the nested PCR. The fragment size of the obtained sequences ranged from 509 to 600 bp. Eighty-four sequences from this study showed 100% identity with each other whereas one sequence showed 99.6% nucleotide identity with the other sequences from this study. BLAST analysis of the 600 bp region of 16 S rRNA gene amplicon of one representative sequence out of the 84 identical sequences from this study (named CMhl alpaca Italy 1 2021) showed nucleotide identity ranging from 97.8% (accession number FN908077) to 100% (accession numbers JF495171 and GU047355) with CMhl sequences deposited in GenBank (query coverage 100% and E-value 0.0 for both). The other sequence of this study (named CMhl alpaca Italy 2 2023) showed 100% nucleotide identity with CMhl strain Purdue (accession numbers CP003731; query coverage 100% and E-value 0.0) and a CMhl sequence from Ohio (accession number FJ527244; (query coverage 99% and E-value 0.0) deposited in GenBank. The distribution of the haplotypes in positive herds is reported in Fig. 1.

**Fig. 3:**
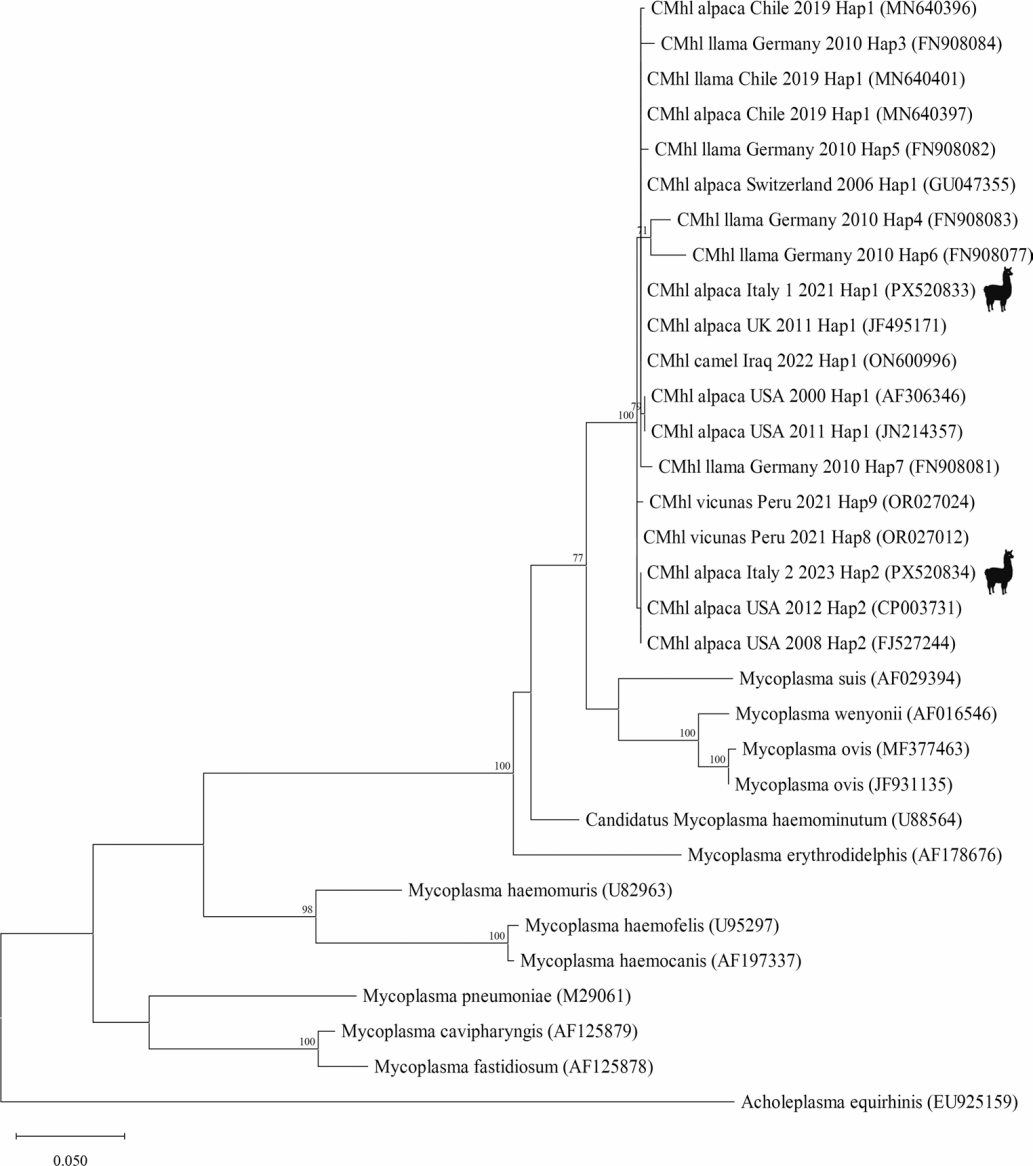
Phylogenetic tree generated with ML analysis. A 600 bp region of 16 S rRNA gene obtained from *Candidatus* Mycoplasma haemolamae and other *Mycoplasma* spp. sequences retrieved from GenBank databases and the CMhl representative sequences obtained in this study were used. Sequences are indicated by *Mycoplasma* species and GenBank accession number (available at http://www.ncbi.nlm.nih.gov/pubmed/) and for CMhl sequences animal species, Country, year of collection, haplotype (as reported by Ramos et al. 2021) are also indicated. For the purpose of this study, the two additional CMhl haplotypes reported in vicunas from Peru have been identified as CMhl haplotype#8 and haplotype#9. TN93 (Tamura and Nei 1993) and gamma distribution with invariant sites (G + I) model were used as parameters of nucleotide substitution, and the analytical procedure encompassed 30 nucleotide sequences with 612 positions in the final dataset. Bootstrap values above 70% are given. The two representative sequences obtained in this study are shown

Phylogeny based on ML analysis showed that the two representative sequences obtained from alpacas and llama from this study clustered with CMhl sequences retrieved from GenBank (Fig. 3).

Based on sequence analysis, the representative sequence for the 84 identical sequences of this study belonged to CMhl haplotype #1 whereas the other representative sequence from one animal of this study belonged to CMhl haplotype #2. CMhl haplotype #1 was found in all positive herds. One herd from Northern Italy also showed the presence of CMhl haplotype #2 (Fig. 1, Table S2).

### Factors associated with CMhl positivity

Results of statistical analysis based on Pearson chi-square test or Fisher’s exact tests to evaluate the differences between proportions of CMhl-positive animals and variables are reported in Table 1. Age categories, herd size, area of origin, season, and health status were significantly associated with CMhl positivity. Regarding age categories, cria significantly showed the lowest CMhl positivity compared to weaners (*P* < 0.001), yealings (*P* = 0.01) and adults (*P* = 0.0178). Moreover, weaners showed a significantly higher CMhl positivity compared to adults (*P* = 0.0263) but similar to yearlings (*P* = 0.21). Yearlings and adults showed similar CMhl positivity (*P* = 0.4446).

No correlation was detected between CMhl positivity and the presence of haematological changes (Table 1). Out of the 59 alpacas with haematological analysis, 22 were CMhl-positive and 37 CMhl-negative. Haematological changes were observed in both CMhl-positive (9/22, 40.9%) and CMhl-negative animals (8/37, 21.6%). More precisely, the nine CMhl-positive animals showed haematological changes including thrombocytopenia (*n* = 3), anemia (*n* = 2) haemoconcentration (*n* = 1), leukocytosis (*n* = 1), and two animals showed both anemia and leukocytosis. The majority (13/22, 59.1%) of CMhl-positive animals did not show haematological changes.

Results of univariable analysis are reported in Table S4. Binary logistic regression analysis showed the association of the dependent variable (probability of CMhl-positive result) with some of the independent variables (Table 3), confirming some of the variables based on Pearson chi-square test or Fisher’s exact tests to evaluate the differences between proportions of CMhl-positive animals.

**Table 3:** Binary logistic regression analysis for association of variables with CMhl positivity

Variable	*P* value*	OR (95% CI)
Sex	*0.062*	1.772 (0.972–3.233)
Herd size	**0.011**	2.136 (1.190–3.834)
Area	**0.031**	0.569 (0.341–0.949)
Season	**0.003**	0.529 (0.347–0.806)1

* values in bold are statistically significant, *P* < 0.05; values in italics are tendencies, *P* < 0.1; CI- confidence interval; OR – odds ratio

Herd size showed a significant effect on CMhl positivity showing the increase of the herd size (from small to large) as a risk factor for CMhl positivity. The probability of a CMhl-positive result was more than doubled from small to medium and from medium to large herds, suggesting higher risk of CMhl-positivity in large herds.

The geographical area of origin was significantly associated with CMhl positivity, showing a decrease of positivity from North to South Italy. These result show that the risk of being CMhl-positive is related to the geographical area of origin, with a higher risk in Northern Italy. A significant seasonal effect on CMhl positivity was observed, with positivity rates decreasing from warmer to colder months.

Results showed that summer is associated with a higher CMhl-positivity, followed by autumn.

The goodness of fit of the model evaluated with Hosmer and Lemeshow test (Chi-square = 4.448; df = 7; *P* = 0.727) showed the absence of statistical difference between the observed results and those predicted by the model, confirming the good representation of the observed data by the model used and the accuracy of the estimated probabilities.

No significant differences were found in the CMhl loads in positive animals according to sex, age and health status. Higher CMhl mean loads tended to be associated with unhealthy compared to healthy status of animals (*P* = 0.065).

Concordance between blood smear and qPCR for the detection of CMhl is reported in Table 4. A Cohen Kappa measure of 0.4687 was calculated, corresponding to a moderate concordance between the two tests. Sensitivity and specificity of blood smear compared to qPCR were 42.9% and 100%, respectively.

**Table 4:** Concordance between blood smear and qPCR for the detection of CMhl

		qPCR	qPCR	qPCR
		positive	negative	Total
Blood smear	positive	6	0	6
	negative	8	20	28
Total		1	20	34

## Discussion

*‘Candidatus* M. haemolamae’ has previously been described as an endemic pathogen of camelids, causing either subclinical infections or clinical manifestations, such as hemolytic anemia. Reports of CMhl in alpacas and llamas have been documented in North and South America (Lascola et al. 2009; Tornquist et al. 2010; Almy et al. 2006; Ramos et al. 2021; Gomez-Puerta et al. 2024), Europe (Kaufmann et al. 2011; Crosse et al. 2012; Franz et al. 2016; Wagener et al. 2024) and New Zealand (van Andel et al. 2013; Dittmer et al. 2018). This study investigated CMhl infection in Italy, providing evidence that CMhl is widespread in alpacas and llamas throughout the country.

As previously reported, our results confirm that blood smear analysis presents low sensitivity for diagnosing CMhl infection. Interpretation of blood smear should not rely on the moderate agreement indicated by Cohen’s Kappa, which likely reflects the paradoxical behavior of this analysis in the presence of strong prevalence or bias effects. In such situations, Kappa values may underestimate the true level of agreement between diagnostic tests, even when overall concordance is high, limiting the interpretation of results (Feinstein and Cicchetti 1990). Overall, the diagnostic limitations of blood smear, even if widely used, suggest that blood smear should not be used for CMhl diagnosis, and qPCR test should instead be carried out to confirm or rule out an infection (Wagener et al. 2024). Moreover, the detection of CMhl using qPCR analysis in three animals with low CMhl loads that were negative by nested PCR for sequencing, confirms previous studies indicating that qPCR is more sensitive than conventional PCR, when CMhl loads are low (Meli et al. 2010; Collere et al. 2025).

The overall positivity detected in our study (42.7%) by qPCR analysis may have been influenced by the convenience sampling of this study, as animals were also recruited for diagnostic purposes, and it does not allow us to infer CMhl prevalence in the population of alpacas and llamas in Italy. A possible overestimation bias due to this type of sampling may have occurred.

However, the overall positivity detected herein is consistent with recent reports from Europe on alpacas, including Germany (37.1%) and Finland (35.3%) (Wagener et al. 2024). Similar findings have also been reported in the USA (34.2%) in alpacas and llamas in eastern Tennessee (Viesselmann et al. 2019). In contrast, lower CMhl positivity rates have been reported in other geographical areas, ranging from 0.97% in New Zealand to 29% in the UK in SAC (McLaughlin et al. 1990; Kaufmann et al. 2010; Tornquist et al. 2010; Kaufmann et al. 2011; Franz et al. 2016; Dittmer et al. 2018; Ramos et al. 2021; Gomez-Puerta et al. 2024). Moreover, the variability in farm-level positivity in this study agrees with previous reports, which described higher CMhl prevalence in some farms compared to others (Meli et al. 2010; Crosse et al. 2012; Pentecost et al. 2012), reaching up to 100% (Viesselmann et al. 2019). Among the risk factors for CMhl infection, the association between larger herd size and higher numbers of CMhl-positive animals was expected, as higher animal density generally enhances the likelihood of pathogen transmission within the group (Gilchrist et al. 2007). On the other hand, herd size alone does not explain the differences in positivity when comparing different countries and regions. Differences in CMhl positivity among countries may be also influenced variations in diagnostic approaches. Although qPCR has been employed in studies reporting both high and very low CMhl positivity (Dittmer et al. 2018), a direct comparison between the qPCR method used in the present study and those applied in previous research was not performed, which may have impacted the observed positivity rates. Variations in CMhl prevalence may also be affected by geographical and climate differences (Franz et al. 2016; Viesselmann et al. 2019). The subtropical latitude and the high temperatures have been suggested as factors associated with CMhl positivity for camelids farmed in Tennessee, USA (Viesselmann et al. 2019). These factors may similarly explain the overall high positivity of CMhl in Italy, and the differences across the country. Indeed, climate changes are contributing to high temperatures across Italy, including in the North (Bionda et al. 2024). However, Southern Italy is usually characterized by higher temperatures compared to the North of the country and the lower positivity in animals from Southern Italy may be biased, as all animals from that area belonged to small herds and were sampled in spring and autumn, both factors associated with lower CMhl positivity.

Seasonal variability, with higher CMhl positivity during warmer months, was expected and confirmed, as previously reported in the USA (Tornquist et al. 2010), even if the limited number of animals sampled in summer may have influenced the results. Warmer temperatures are likely to promote vector-borne disease transmission and suggest a potential role for arthropod vectors in CMhl transmission. Indeed, although the biological or mechanical putative vectors of CMhl are not yet known, it has been suggested that biting arthropods may transmit CMhl, as for other hemoplasmosis (Ramos et al. 2021). Recently, SAC ticks (*Amblyomma parvitarsum*) have been suggested to play a role in the transmission of CMhl in vicunas in Peru (Gomez-Puerta et al. 2024). Variations in vectors have been also proposed to be associated with the differences in prevalence found in different countries, regions and farms (Franz et al. 2016). Further studies are needed to clarify the role of vectors in the transmission of CMhl in Italy, also considering that, even if ticks were not observed on study animals at sampling time, prior exposure to vectors cannot be ruled out. Unfortunately, information was not collected to define if tick infestation has been previously reported in the alpacas and llamas included in this study, nor about the tick species that may have parasitized them. However, recent surveys investigating tick occurrence in the same regions of Italy where the study alpaca farms were located reported that the most commonly reported tick species in Italy include *Rhipicephalus sanguineus*,* Rhipicephalus bursa*, *Dermacentor marginatus*, *Dermacentor reticulatus*, *Ixodes canisuga*, *Ixodes hexagonus*, *Ixodes ricinus*, and *Haemaphysalis punctata*, as reported in a study on dogs (Zanet et al. 2020). In addition, *Rhipicephalus turanicus* has been detected in wild boars in southern Italy (Sgroi et al. 2022).

In addition to warm temperatures, other factors may be associated with the overall high positivity observed in our study, especially considering that similar positivity has been observed in colder countries such as Germany and Finland (Wagener et al. 2024). Among these factors, age may also play a role in CMhl positivity. Although this association was not confirmed by binary logistic regression (likely due to the limited number of animals in certain age categories), chi square results seem to suggest that younger animals may be at increased risk of CMhl infection. The significant association observed in the chi-square analysis should be considered indicative only, given the unbalanced distribution of animals across categories, and the inability of this test to adjust for potential confounders. Therefore, this result cannot be interpreted as evidence of a true causal relationship and requires confirmation in future studies with larger and more balanced groups. However, the finding of this study is in agreement with other studies identifying younger age as a risk factor for CMhl infection (McLaughlin et al. 1990), but contrasts with others showing older age or no association of age with infection (Tornquist et al. 2010; Kaufmann et al. 2011). The significantly higher CMhl positivity in weaners compared to cria suggests that weaning (around six months) may represent a critical period for infection in alpacas. This may be associated with decline in maternal antibodies derived by passive immunity transfer via colostrum. However, passive immunity transfer in alpacas has been reported to last for several weeks to few months and in most animals the decline in serum antibodies has been reported to cease after 42 days of age, indicating activity of cria’s immune system (Daley-Bauer et al. 2010). Therefore, additional factors may contribute to the higher CMhl-positivity in weaners, potentially including differences in camelid husbandry practices (e.g., management of weaning, housing density, hygiene, pasture rotation, or tick control measures), which, however, were not investigated in the present study. On the other hand, the protection due to passive immunity transfer via maternal colostrum (Pentecost et al. 2012) may explain the very low level of CMhl positivity in crias of this study. Indeed, the only positive cria did not receive maternal colostrum. Therefore, considering that alpacas have an epitheliochorial placenta and crias are born agammaglobulinemic, achievement of adequate passive transfer of immunity by colostral transfer in crias should always be monitored to reduce the risk of infectious diseases (Wernery 2001).

Additionally, our findings are consistent with previous reports indicating that sex is not a risk factor for CMhl positivity (Tornquist et al. 2010).

Regarding the clinical signs observed in our study group related to the pathogenicity of CMhl, our results indicated a higher prevalence of positive cases in the non-healthy group; however, this expectation was not corroborated by the binary logistic regression analysis. Although the significant association observed in the chi-square analysis should be considered indicative only and requires further investigation before being confirmed, the higher prevalence of positive cases in the non-healthy group was expected, given that CMhl infects erythrocytes and has been linked to anemia (Lascola et al. 2009; McLaughlin et al. 1990; Meli et al. 2010; Tornquist et al. 2010), which was one of the characteristics for the unhealthy group. Notably, unhealthy status was also noted in CMhl-negative animals; thus, clinical manifestations in these instances might be attributed to other uninvestigated morbidities.

When considering the total number of CMhl-infected alpacas and llamas in this study, it has to be considered that most CMhl-infected animals appeared clinically healthy and often exhibit no hematological alterations, suggesting low pathogenicity of CMhl, in accordance with previous reports reporting majority of infected animals as asymptomatic carriers that never develop clinical disease (Ramos et al. 2021; Wagener et al. 2024). Understanding the mechanisms underlying the seemingly low pathogenicity of CMhl is crucial, and there is speculation that the unique characteristics of camelid immunity may render them less susceptible to various pathogens (Larska et al. 2009). Overall, our findings suggest that antimicrobial treatment is likely not warranted based on qPCR positivity alone (Viesselmann et al. 2019).

No information regarding the genetic diversity of CMhl in Italy was previously available and this is the first study that addressed the haplotype diversity of CMhl. The characterization of CMhl haplotypes showing that the majority of positive samples belonged to haplotype #1 confirms this haplotype as the most frequent worldwide (Meli et al. 2010; Ramos et al. 2021), that has been previously reported in North America (USA), South America (Chile), Europe (UK, Germany, Switzerland) and Iraq based on the presence of available sequences deposited in GenBank (Ramos et al. 2021). Moreover the presence of another haplotype (CMhl haplotype #2), previously reported in USA and Germany, was identified in one herd from Italy. This result highlights the possible circulation of different CMhl haplotypes in some countries. The presence of two different CMhl haplotypes has been reported in USA in alpacas and llamas and in Peru in vicunas (Ramos et al. 2021; Gomez-Puerta el al., 2024), whereas seven different CMhl haplotypes have been previously reported in Germany where positive herds had history of recurrent introduction of new animals from other European and non-European countries (Ramos et a., 2021). Unfortunately, origin of animals was not investigated in this study. However, in the specific case of the alpaca positive for CMhl haplotype #2, it was born and raised in Italy. Moreover, potential difference in haplotypes pathogenicity should be clarified.

The transmission routes of CMhl still remain unknown. The results of our study showing the presence of a CMhl-positive cria in the absence of colostral transfer likely rules out colostral transmission of CMhl from the mother (Pentecost et al. 2012). Although the only CMhl-positive cria was born from a CMhl-positive mother, the low CMhl positivity detected in crias in our study suggests that vertical *in utero* transmission is an unlikely or infrequent transmission route. Given that we did not collect information on the mother of the CMhl-negative crias, future studies are needed on mothers and crias with a significant sample size to confirm possible *in utero* transmission, as previously suggested (Pentecost et al. 2012).

This study had several limitations. Data on origin of animals were not recorded, and importation of animals was not investigated in this study as a risk factor for CMhl positivity. However, similar CMhl-positivity has been observed in both indigenous and imported alpacas and llamas from South America (Kaufman et al., 2010; Meli et al. 2010; Crosse et al. 2012), except in one study showing that imported animals were more frequently positive (Kaufmann et al. 2011). Data on antimicrobial therapy history, that may have influenced CMhl positivity results, were not recorded for the purpose of this study. Another limitation is the lack of investigation on persistence of CMhl-positivity that may have added information on CMhl epidemiology.

In this study, only a fragment of the conserved 16 S rRNA gene was sequenced for haplotype identification and the genetic diversity of the CMhl strains detected in this study may have been underestimated. Further studies based on other genes such as the *rnpB* gene (Peters et al. 2008), the full 16 S rRNA gene, other less conserved loci, including the 23 S rRNA (Mongruel et al. 2020), or whole genome sequencing are needed to fully characterize the genetic features of CMhl strains circulating in Italy.

## Conclusion

Our results, showing the presence of CMhl haplotype #1 and haplotype #2 in SAC from Italy, likely associated with large herd size and warm season, highlight the need for further studies on the genetic features, epidemiology, and immune response of CMhl-positive SAC in Italy.

## Supplementary information

Below is the link to the electronic supplementary material.

Supplementary File 1 (DOCX 23.0 KB)

Supplementary File 2 (DOCX 23.2 KB)

Supplementary File 3 (DOCX 17.3 KB)

Supplementary File 4 (DOCX 25.0 KB)

## Data Availability

No datasets were generated or analysed during the current study.
