# Supplementary material for: Detection and characterization of Candidatus mycoplasma haemolamae haplotype in South American camelids farmed in Italy
Source: Vet Res Commun. 2026 Jan 20;50(2):113. doi: 10.1007/s11259-025-11033-y (PMC12819477; doi:10.1007/s11259-025-11033-y)
Supplement: Supplementary file 1 — (DOCX 23.0 KB) [file 11259_2025_11033_MOESM1_ESM.docx]

Research Veterinary Communications

Detection and characterization of *Candidatus* Mycoplasma haemolamae haplotype in South American Camelids farmed in Italy

Stefania Lauzi^a^, Elisa Castaldo^c^, Gabriele Ratti^a^, Giulia Sala^b^, Alessandra Cafiso^a^, Alessia Facchin^a^, Joel Filipe^a^, Donatella Scavone^a^, Cristina Crespi^a^, Stefano Scarcelli^c^, Laura Filippone Pavesi^a^, Camilla Luzzago^a*^, Antonio Boccardo^a^, Davide Pravettoni^a^, Vincenzo Veneziano^c^, Alessia Giordano^a^

*^a^Department of Veterinary Medicine and Animal Sciences, University of Milan, Via dell’Università 6, 26900 Lodi, Italy*

*^b^Department of Veterinary Science, University of Pisa, via Livornese s.n.c, 56122, San Piero a Grado, Italy*

*^c^Department of Veterinary Medicine and Animal Production, University of Naples Federico II, Via Federico Delpino, 1, 80137 Naples, Italy*

* Corresponding author: Camilla Luzzago

*E-mail address*: camilla.luzzago@unimi.it

**Table S1** Amplification specifications (temperature and cycling conditions) of the PCR protocols and qPCR assay from this study.

| **Assay** | **PCR/qPCR protocol** | **Cycle step** | **Temperature** | **Time** | **Cycles** |
| --- | --- | --- | --- | --- | --- |
| Plasmids | PCR CMhl 16S rRNA (600bp)* | Initial denaturation | 98°C | 30s | 1x |
|  |  | Denaturation | 98°C | 5s |  |
|  |  | Annealing | 61°C | 5s | 40x |
|  |  | Extension | 72°C | 10s |  |
|  |  | Final extension | 72°C | 1min |  |
|  | PCR CMhl 16S rRNA (72bp) | Initial denaturation | 95°C | 2min | 1x |
|  |  | Denaturation | 98°C | 40s |  |
|  |  | Annealing | 60°C | 45s | 40x |
|  |  | Extension | 72°C | 45s |  |
|  |  | Final extension | 72°C | 5min |  |
| CMhl detection | qPCR CMhl 16S rRNA (72bp) | UNG activation | 50°C | 2min | 1x |
|  |  | Initial denaturation | 95°C | 2min | 1x |
|  |  | Denaturation | 95°C | 15s | 40x |
|  |  | Extension | 60°C | 1min |  |
|  |  | Melt curve** |  |  |  |
| CMhl haplotype | PCR CMhl 16s rRNA (1420bp)- outer reaction | Initial denaturation | 98°C | 30s | 1x |
|  |  | Denaturation | 98°C | 5s |  |
|  |  | Annealing | 56°C | 5s | 40x |
|  |  | Extension | 72°C | 20s |  |
|  |  | Final extension | 72°C | 1min |  |
|  | PCR CMhl 16S rRNA (600bp)- inner reaction* | Initial denaturation | 98°C | 30s | 1x |
|  |  | Denaturation | 98°C | 5s |  |
|  |  | Annealing | 61°C | 5s | 40x |
|  |  | Extension | 72°C | 10s |  |
|  |  | Final extension | 72°C | 1min |  |

* same PCR protocol; **a melting curve was determined with a transition rate of 0.5°C/s from 60°C to 95°C. Melting peaks were automatically calculated by QuantStudio™ Design & Analysis Software v1.4.3 (Thermo Fisher Scientific, Waltham, MA, USA).
