# Supplementary material for: Detection and characterization of Candidatus mycoplasma haemolamae haplotype in South American camelids farmed in Italy
Source: Vet Res Commun. 2026 Jan 20;50(2):113. doi: 10.1007/s11259-025-11033-y (PMC12819477; doi:10.1007/s11259-025-11033-y)
Supplement: Supplementary file 2 — (DOCX 23.2 KB) [file 11259_2025_11033_MOESM2_ESM.docx]

Research Veterinary Communications

Detection and characterization of *Candidatus* Mycoplasma haemolamae haplotype in South American Camelids farmed in Italy

Stefania Lauzi^a^, Elisa Castaldo^c^, Gabriele Ratti^a^, Giulia Sala^b^, Alessandra Cafiso^a^, Alessia Facchin^a^, Joel Filipe^a^, Donatella Scavone^a^, Cristina Crespi^a^, Stefano Scarcelli^c^, Laura Filippone Pavesi^a^, Camilla Luzzago^a*^, Antonio Boccardo^a^, Davide Pravettoni^a^, Vincenzo Veneziano^c^, Alessia Giordano^a^

*^a^Department of Veterinary Medicine and Animal Sciences, University of Milan, Via dell’Università 6, 26900 Lodi, Italy*

*^b^Department of Veterinary Science, University of Pisa, via Livornese s.n.c, 56122, San Piero a Grado, Italy*

*^c^Department of Veterinary Medicine and Animal Production, University of Naples Federico II, Via Federico Delpino, 1, 80137 Naples, Italy*

* Corresponding author: Camilla Luzzago

*E-mail address*: camilla.luzzago@unimi.it

**Table S2** Number and percentage of CMhl-positive alpaca and llama tested in this study according to farm of origin.

| Farm | Area* | Total animals | No. tested (%) | No. CMhl-positive (%) | No. CMhl haplotype #1 positive animals | No. CMhl haplotype #2 positive animals |
| --- | --- | --- | --- | --- | --- | --- |
| 1 | C | 300 | 8 (2.7) | 7 (87.5) | 7 | - |
| 2 | N | 6 | 4 (66.7) | 2 (50) | 2 | - |
| 3 | N | 7 | 5 (71.4) | 2 (40) | 1 | 1 |
| 4 | N | 23 | 4 (17.4) | 0 (0) | - | - |
| 5 | C | 6 | 5 (83.3) | 4 (80) | 4 | - |
| 6 | S | 4 | 4 (100) | 0 (0) | - | - |
| 7 | C | 9 | 5 (55.6) | 3 (60) | 3 | - |
| 8 | C | 40 | 8 (20) | 5 (62.5) | 5 | - |
| 9 | N | 38 | 4 (10.5) | 4 (100) | 4 | - |
| 10 | N | 20 | 4 (20) | 2 (50) | 2 | - |
| 11 | N | 28 | 5 (17.9) | 5 (100) | 5 | - |
| 12 | N | 3 | 2 (66.7) | 0 (0) | - | - |
| 13 | C | 13 | 4 (30.8) | 4 (100) | 4 | - |
| 14 | S | 5 | 4 (80) | 0 (0) | - | - |
| 15 | C | 4 | 4 (100) | 0 (0) | - | - |
| 16 | S | 2 | 2 (100) | 2 (100) | 2 | - |
| 17 | N | 22 | 4 (18.2) | 2 (50) | 2 | - |
| 18 | N | 23 | 4 (17.4) | 2 (50) | 2 | - |
| 19 | C | 2 | 2 (100) | 0 (0) | - | - |
| 20 | C | 2 | 1 (50) | 0 (0) | - | - |
| 21 | N | 8 | 4 (50) | 0 (0) | - | - |
| 22 | S | 14 | 6 (42.9) | 0 (0) | - | - |
| 23 | N | 38 | 4 (10.5) | 3 (75) | 3 | - |
| 24 | N | 7 | 2 (28.6) | 0 (0) | - | - |
| 25 | C | 7 | 1 (14.3) | 0 (0) | - | - |
| 26 | N | 17 | 17 (100) | 12 (70.6) | 12 | - |
| 27 | N | 2 | 1 (50) | 0 (0) | - | - |
| 28 | N | 7 | 7 (100) | 4 (57.1) | 4 | - |
| 29 | N | 12 | 12 (100) | 1 (8.3) | 1 | - |
| 30 | C | 27 | 27 (100) | 10 (37) | 10 | - |
| 31 | N | 7 | 7 (100) | 2 (28.6) | 2 | - |
| 32 | N | 12 | 12 (100) | 1 (8.3) | 1 | - |
| 33** | N | 13 | 13 (100) | 9 (69.2) | 9 | - |
| 34 | C | 40 | 1 (2.5) | 1 (nd) | 1 | - |
| 35 | C | 9 | 9 (100) | 1 (11.1) | 1 | - |

* C= Center Italy, N= North Italy, S= South Italy; ** herd with alpaca and llama; nd: not determined
