# Supplementary material for: Detection and characterization of Candidatus mycoplasma haemolamae haplotype in South American camelids farmed in Italy
Source: Vet Res Commun. 2026 Jan 20;50(2):113. doi: 10.1007/s11259-025-11033-y (PMC12819477; doi:10.1007/s11259-025-11033-y)
Supplement: Supplementary file 3 — (DOCX 17.3 KB) [file 11259_2025_11033_MOESM3_ESM.docx]

Research Veterinary Communications

Detection and characterization of *Candidatus* Mycoplasma haemolamae haplotype in South American Camelids farmed in Italy

Stefania Lauzi^a^, Elisa Castaldo^c^, Gabriele Ratti^a^, Giulia Sala^b^, Alessandra Cafiso^a^, Alessia Facchin^a^, Joel Filipe^a^, Donatella Scavone^a^, Cristina Crespi^a^, Stefano Scarcelli^c^, Laura Filippone Pavesi^a^, Camilla Luzzago^a*^, Antonio Boccardo^a^, Davide Pravettoni^a^, Vincenzo Veneziano^c^, Alessia Giordano^a^

*^a^Department of Veterinary Medicine and Animal Sciences, University of Milan, Via dell’Università 6, 26900 Lodi, Italy*

*^b^Department of Veterinary Science, University of Pisa, via Livornese s.n.c, 56122, San Piero a Grado, Italy*

*^c^Department of Veterinary Medicine and Animal Production, University of Naples Federico II, Via Federico Delpino, 1, 80137 Naples, Italy*

* Corresponding author: Camilla Luzzago

*E-mail address*: camilla.luzzago@unimi.it

**Table S3** Mean and median CMhl-loads (DNA copy/µL blood) in CMhl-positive alpacas and llamas according to characteristics of animals.

| Variable | Category | Mean CMhl-load ± SD* | median CMhl load | min | max |
| --- | --- | --- | --- | --- | --- |
| Sex | female | 3.7 x 10^5^ ±1.3 x 10^6^ | 2.8 x 10^4^ | 2,4 x 10^1^ | 2.9 x 10^6^ |
|  | male | 9.7.4 x 10^4^ ±1.5 x 10^5^ | 2.3 x 10^4^ | 2,9 x 10^1^ | 1.9 x 10^9^ |
| Age | crias (<6 months)** | 1.9 x 10^9^ | 1.9 x 10^9^ | nd | nd |
|  | weaner (≥6 months- to <1 year) | 3.8 x 10^5^ ±8.6 x 10^5^ | 9.8 x 10^4^ | 2,4 x 10^1^ | 2.9 x 10^6^ |
|  | tuis (≥1 year to <2 years) | 5.6 x 10^5^ ±1.7 x 10^6^ | 6.7 x 10^4^ | 5.7 x 10^3^ | 7.2 x 10^6^ |
|  | adult (≥ 2 years) | 1.2 x 10^5^ ±4.4 x 10^5^ | 4.7 x 10^3^ | 8 | 2.9 x 10^6^ |
| Health status | clinically healthy | 3.8 x 10^5^ ±1.2 x 10^6^ | 2.7 x 10^4^ | 2,4 x 10^1^ | 2.9 x 10^6^ |
|  | clinically unhealthy | 1.9 x 10^8^ ±6 x 10^8^ | 8 x 10^3^ | 2,9 x 10^1^ | 1.9 x 10^9^ |

*standard deviation.

** only one positive animal
