# Supplementary material for: Detection and characterization of Candidatus mycoplasma haemolamae haplotype in South American camelids farmed in Italy
Source: Vet Res Commun. 2026 Jan 20;50(2):113. doi: 10.1007/s11259-025-11033-y (PMC12819477; doi:10.1007/s11259-025-11033-y)
Supplement: Supplementary file 4 — (DOCX 25.0 KB) [file 11259_2025_11033_MOESM4_ESM.docx]

Research Veterinary Communications

Detection and characterization of *Candidatus* Mycoplasma haemolamae haplotype in South American Camelids farmed in Italy

Stefania Lauzi^a^, Elisa Castaldo^c^, Gabriele Ratti^a^, Giulia Sala^b^, Alessandra Cafiso^a^, Alessia Facchin^a^, Joel Filipe^a^, Donatella Scavone^a^, Cristina Crespi^a^, Stefano Scarcelli^c^, Laura Filippone Pavesi^a^, Camilla Luzzago^a*^, Antonio Boccardo^a^, Davide Pravettoni^a^, Vincenzo Veneziano^c^, Alessia Giordano^a^

*^a^Department of Veterinary Medicine and Animal Sciences, University of Milan, Via dell’Università 6, 26900 Lodi, Italy*

*^b^Department of Veterinary Science, University of Pisa, via Livornese s.n.c, 56122, San Piero a Grado, Italy*

*^c^Department of Veterinary Medicine and Animal Production, University of Naples Federico II, Via Federico Delpino, 1, 80137 Naples, Italy*

* Corresponding author: Camilla Luzzago

*E-mail address*: camilla.luzzago@unimi.it

**Table S4** Characteristics of the animals analyzed in this study, including univariate analysis p-values for association with CMhl positivity.

| Variable | Category | No. tested | No. CMhl-positive (%) | *P* value* |
| --- | --- | --- | --- | --- |
| Species | alpaca | 200 | 83 (41.5) | 0.206 |
|  | llama | 6 | 5 (83.3) |  |
| Sex | male | 103 | 49 (47.6) | 0.257 |
|  | female | 103 | 39 (37.9) |  |
| Age | crias (< 6 months) | 13 | 1 (7.7) | 0.552 |
|  | weaner (≥ 6 months to < 1 year) | 15 | 11 (73.3) |  |
|  | tuis (≥ 1 year to < 2 years) | 34 | 17 (50) |  |
|  | adult (≥ 2 years) | 144 | 59 (41) |  |
| Herd size | small (≤ 20) | 137 | 49 (35.8) | **0.068** |
|  | medium (> 20 to ≤50) | 61 | 32 (52.5) |  |
|  | large (> 50) | 8 | 7 (87.5) |  |
| Area | North | 75 | 35 (46.7) | **0.139** |
|  | Center | 115 | 51 (44.3) |  |
|  | South | 16 | 2 (12.5) |  |
| Season | spring | 70 | 19 (27.1) | **<.001** |
|  | summer | 4 | 3 (75) |  |
|  | autumn | 37 | 22 (59.5) |  |
|  | winter | 95 | 44 (46.3) |  |
| Health status** | clinically healthy | 111 | 40 (36.0) | **0.034** |
|  | clinically unhealthy | 14 | 10 (71.4) |  |
| Haematology*** | alteration | 17 | 9 (52.9) | 0.288 |
|  | normal | 42 | 13 (31) |  |
| Anemia*** | yes | 6 | 4 (66.7) | 0.128 |
|  | no | 53 | 18 (34) |  |
| Leukocytosis*** | yes | 5 | 3 (60) | 0.438 |
|  | no | 54 | 19 (35.2) |  |
| Haemoconcentration*** | yes | 3 | 1 (33.3) | 1 |
|  | no | 56 | 21 (37.5) |  |
| Thrombocytopenia*** | yes | 4 | 3 (75) | 0.438 |
|  | no | 55 | 19 (34.5) |  |

** Health status was available for 125 animals.

**Haematological parameters were available for 59 alpacas.
